# ViTAL: Vision TrAnsformer based Low coverage SARS-CoV-2 lineage assignment

**DOI:** 10.1093/bioinformatics/btae093

**Published:** 2024-02-19

**Authors:** Zuher Jahshan, Leonid Yavits

**Affiliations:** EnICS Labs, Engineering Department, Bar-Ilan University, Ramat Gan, Tel Aviv 5290002, Israel; EnICS Labs, Engineering Department, Bar-Ilan University, Ramat Gan, Tel Aviv 5290002, Israel

## Abstract

**Motivation:**

Rapid spread of viral diseases such as Coronavirus disease 2019 (COVID-19) highlights an urgent need for efficient surveillance of virus mutation and transmission dynamics, which requires fast, inexpensive and accurate viral lineage assignment. The first two goals might be achieved through low-coverage whole-genome sequencing (LC-WGS) which enables rapid genome sequencing at scale and at reduced costs. Unfortunately, LC-WGS significantly diminishes the genomic details, rendering accurate lineage assignment very challenging.

**Results:**

We present ViTAL, a novel deep learning algorithm specifically designed to perform lineage assignment of low coverage-sequenced genomes. ViTAL utilizes a combination of MinHash for genomic feature extraction and Vision Transformer for fine-grain genome classification and lineage assignment. We show that ViTAL outperforms state-of-the-art tools across diverse coverage levels, reaching up to 87.7% lineage assignment accuracy at 1× coverage where state-of-the-art tools such as UShER and Kraken2 achieve the accuracy of 5.4% and 27.4% respectively. ViTAL achieves comparable accuracy results with up to 8× lower coverage than state-of-the-art tools. We explore ViTAL’s ability to identify the lineages of novel genomes, i.e. genomes the Vision Transformer was not trained on. We show how ViTAL can be applied to preliminary phylogenetic placement of novel variants.

**Availability and implementation:**

The data underlying this article are available in https://github.com/zuherJahshan/vital and can be accessed with 10.5281/zenodo.10688110.

## 1 Introduction

One of the lessons of COVID-19 pandemic is that our computational infrastructure and bioinformatics tools are inefficient in tracking quickly mutating viral pathogens spread throughout the world. Without a timely and accurate understanding of the transmission dynamics of the pathogen, efficient control of the pandemic is challenging. The foremost tasks of such pathogen tracking are pathogen detection, its assignment into one of the many existing lineages/clades, and discovery of emerging mutations. Lineage assignment is different from genome classification where an organism is classified into one of a few relatively distinct classes of species [such as human versus virus (Acera Mateos *et al.* 2021)]. ViTAL lineage assignment targets hundreds, potentially thousands of very closely related families, which requires very precise fine-grain classification.

Existing diagnostic tools, such as polymerase chain reaction (PCR) tests, can neither detect new variants of a rapidly mutating virus nor efficiently classify and assign viral samples to lineages (Dunn *et al.* 2021). This is because primers must be specifically designed for each new variant. Identifying a new viral sample with one of hundreds of existing variants using PCR is infeasible. A practical solution requires DNA sequencing and digital genome analysis.

Although digital analysis can be relatively quick, assuming the availability of high-performance computing resources, DNA sequencing is a lengthy and expensive process. State-of-the-art Severe acute respiratory syndrome coronavirus 2 (SARS-CoV-2) sequencing targets at least 10× coverage (Sequencing coverage refers to the average number of times each base in the genome is read during the sequencing process.) for 95% of SARS-CoV-2 genomes for clade/lineage assignment, while above 500× coverage is recommended for the determination of minority variants which can contribute significantly to the evidence for direct transmission or reinfection (ECfDPa 2021). These recommendations are generally rigorously followed: a coverage of >1000× is often applied to identify variants of SARS-CoV-2 for genomic surveillance (Goswami *et al.* 2022, Wang *et al.* 2022).

The high coverage recommended for SARS-CoV-2 lineage assignment is resource intensive, time consuming, and expensive. Therefore, it impacts large-scale sequencing efforts and rapid public health responses.

One way to mitigate the cost and time of sequencing is by addressing only a small fraction of samples, such as a representative portion of positive SARS-CoV-2 samples, or a subset of samples of particular interest (e.g. breakthrough cases, reinfections, etc.) However, when only a small portion of the samples are sequenced, there can be several consequences (Quick *et al.* 2016):

- Incomplete picture of virus spread and gaps in our understanding of transmission chains, making it more difficult to implement effective measures to control the spread of the virus.
- Delayed public health response: The speed at which public health measures are implemented can be heavily influenced by the data received from genome sequencing. If there is a delay or lack of data, this can lead to slower responses to outbreaks and potentially higher rates of transmission and death.

In this study, we propose a different way to reduce sequencing costs and time by targeting *low-coverage* sequencing. Reduced coverage requires fewer reads to be generated and processed per sample, leading to higher analysis throughput. Lowering the sequencing coverage without sacrificing accuracy would present a dual advantage of higher throughput and cost-effectiveness [fewer sequencing reagents and less computational resources (Head *et al.* 2014, Schirmer *et al.* 2015)].

Low-coverage whole-genome sequencing (LC-WGS) has recently experienced a surge of interest in the scientific community (Lou *et al.* 2021). Unlike pool sequencing (Ferretti *et al.* 2013), which combines samples and may lose information about their individual origins, LC-WGS preserves the integrity of each sample. It facilitates the simultaneous sequencing of multiple samples, with each sample being sequenced at a significantly lower coverage. This strategy requires only a minimal amount of sequencing per sample, making LC-WGS a cost-effective choice for large-scale studies. As an added advantage, despite the reduction in coverage, the sequencing cost tends to decrease linearly with the exception of sample preparation expenses (Pasaniuc *et al.* 2012). Thus, LC-WGS allows efficient scalability while retaining individual sample information.

In this work, we propose an alternative approach to clade/lineage assignment of SARS-CoV-2 genomes. Our approach is based on two notions. First, we suggest applying the principles of LC-WGS to SARS-CoV-2 lineage assignment, targeting sequencing coverage as low as 1×. Such aggressive coverage reduction is counter-intuitive vis-a-vis the high coverage SARS-CoV-2 sequencing recommendation (ECfDPa 2021). Conventional classification and lineage assignment tools such as Kraken2 and Usher respectively either fail to assign to a clade a genome sequenced with very low coverage, or achieve very limited assignment accuracy (e.g. 27.4%), as shown in Section 3.1.

**Figure 1.**
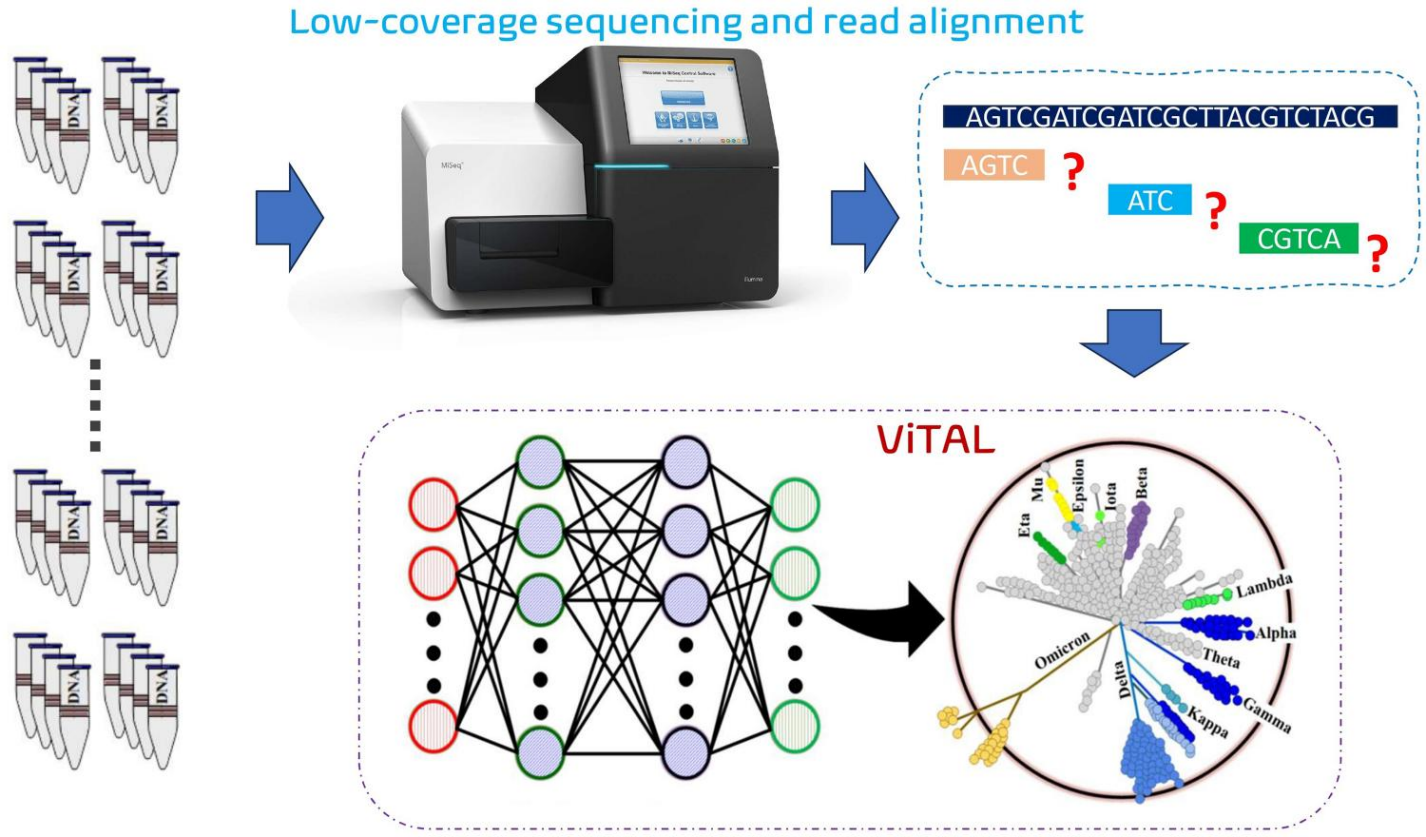
ViTAL: From low-coverage sequencing to high-accuracy viral lineage assignment.

The second notion of our proposal is to move the resolution of the low-accuracy classification problem associated with low-coverage sequencing to the digital domain. Specifically, we develop and apply a novel deep neural network-based algorithm as presented in Fig. 1. It achieves higher lineage assignment accuracy compared to state-of-the-art tools, even when its input genome data has been sequenced with up to 8× lower coverage, as shown in Section 3.1. With the ability to operate efficiently on such low coverage data, we enable the processing of many more viral samples concurrently, thus allowing accurate and timely responses in critical healthcare situations. We hope that our approach will help to democratize the viral genome lineage assignment.

## 2 Materials and methods

ViTAL, the lineage assignment algorithm proposed here, inputs a low-coverage genome, transforms it into embedded genome fragments which are then fed into a classification neural network, that outputs the most likely lineages the input genome might belong to. The ViTAL algorithm is therefore divided into preprocessing phase (MinHash) followed by embedding, and the classification phase (Vision Transformer).

### 2.1 MinHash

**Figure 2.**
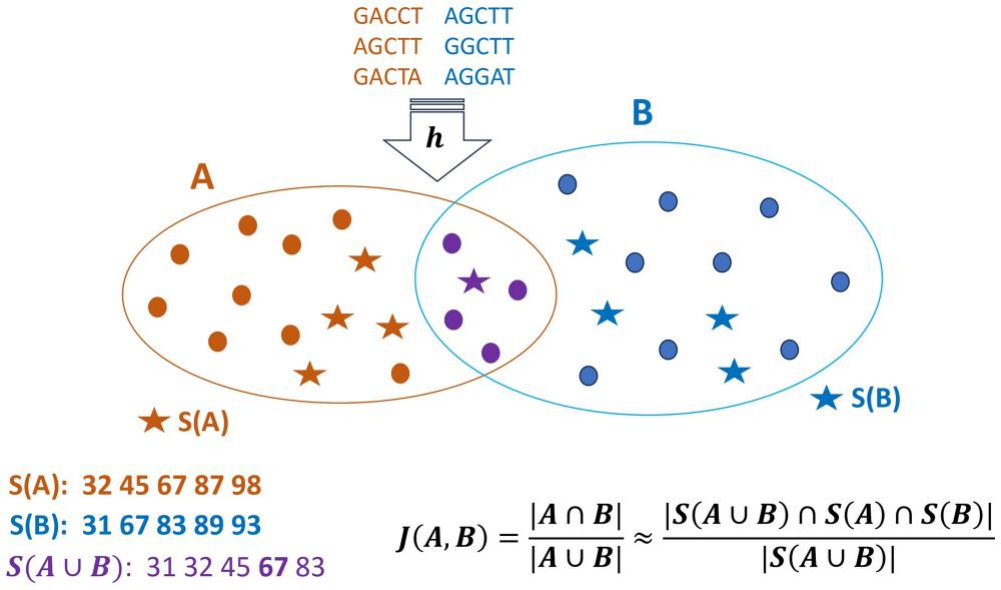
Minhash Bottom-*k* overview [adopted from Ondov *et al.* (2016)].

**MinHash** is a variant of locality sensitive hashing designed to approximate the similarity between two datasets. The foundational idea behind MinHash is that the probability of two sets having the same minimum hash value is equivalent to their Jaccard similarity [Jaccard similarity measures the proportion of shared elements between two sets equal to $J(A,B)=\frac{\left|A\cap B\right|}{\left|A\cup B\right|}$] (Broder 1997, Broder *et al.* 1998). We use the Bottom-*k* Minhash whose overview is presented in Fig. 2. In the genomic context, the datasets comprise sequences of kmers. The signatures *A* and *B* are generated by a single hash function, *h*, which ranks the kmers by assigning a numerical value to each kmer. The *k* minimal ranking values comprise the signature subsets S(A) and S(B). The equation in Fig. 2 approximates Jaccard similarity between the two original kmer sequences.

We leverage Bottom-*k* to convert low coverage genomes into sequences of fixed-size fragments. Genomic samples that come from similar genomes are likely to have overlapping hash values, so that Bottom-*k* is instrumental in capturing their similarity.

### 2.2 Vision transformer

**Transformers** (Vaswani *et al.* 2017) power the large language models and are widely used for natural language processing. Vision Transformer (ViT) (Dosovitskiy *et al.* 2020) is a subcategory of transformers, designed for image classification tasks. While traditional convolutional neural networks (CNNs) process images in a local and hierarchical manner, ViT dissects an image into a series of non-overlapping fixed-size *patches*, linearly embedding each patch into a vector, which are then processed by a series of self-attention mechanisms. In our methodology, we leverage the ViT to process the fixed-size sequences generated from the Bottom-k MinHash transformation of genomes. Although derived from genomic data, these sequences can be treated as patches of the ViT’s standard image processing framework. By doing so, we aim to discern the intricate patterns and dependencies within these sequences, particularly crucial for precise lineage identification in situations of reduced coverage.

**Figure 3.**
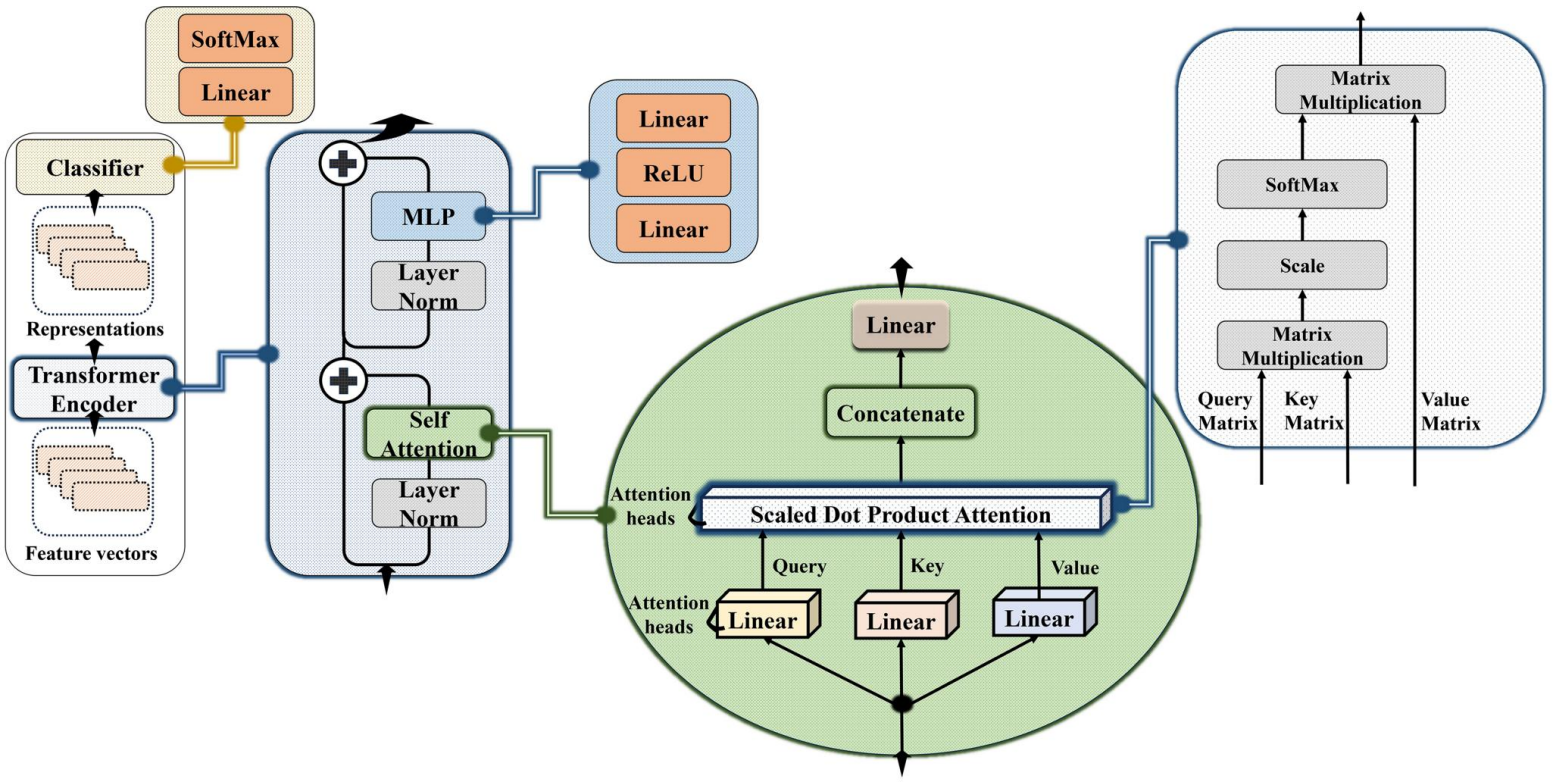
Overview of Vision Transformer comprising Encoder and Classifier; Encoder includes the Self-Attention mechanism, the MLP (a fully connected layer) and several layer normalization steps.

The primary components of ViT, as illustrated in Fig. 3 are:

1. **Stacked Encoder Layers:** The transformer learning ability stems from stacking multiple encoder layers, each comprising the self-attention and other mechanisms described above. This stacking allows for complex inter-patch relationships to be captured.
2. **Self-Attention Mechanism** weighs the *importance* of different patches in relation to each other. Note that an encoder layer typically features several **attention heads** that learn distinct attention representations.
3. **Linear layer** is a fully connected network (followed by an identity activation function).
4. **Query (Q), Key (K), and Value (V)** matrices are derived from each feature vector embedding by passing them through linear layers.
5. **Softmax activation** is used to cap the sum of vector elements at 1 and emphasize the most pertinent vector elements.
6. **Multi-Layer Perceptron (MLP)** in the context of this paper is a fully connected network (a linear layer), followed by a nonlinear activation function, followed by another fully connected layer.
7. **Layer Normalization** is a technique used to bring all input data sources to the same range.
8. **Classifier** is a linear layer followed by a softmax activation, producing the ViT output, which is the set of class probabilities, or in the case of ViTAL, a set of genome lineage assignment probabilities.

In contrast to conventional ViT architecture, positional embeddings [Positional embeddings give first few tens of dimensions of the token embeddings, or relative positional closeness within the input sequence (Vaswani et al. 2017).] are not used in ViTAL due to the unique structure of the input data. Genome data differs from typical 2D images, making standard positional embeddings less efficient or potentially even counterproductive in genome lineage assignment.

### 2.3 Putting it all together

Following are the steps of ViTAL algorithm (Fig. 5).

**1. Pre-processing Phase:**

- MinHash Extraction: Using the MinHash technique, we extract *n* representative k-mers from a given genomic sample.
- Fragment Generation: Every k-mer obtained in the previous step is extended to *f* (where *f* represents the fragment size and f>k). This extension is done to render a representative fragment, with the k-mer positioned in the middle of the fragment (i.e. the k-mer start position is the index $\frac{f-k}{2}$ of the fragment).
- One-Hot Encoding: Each fragment is then transformed using one-hot encoding. The bases are encoded as:
  $$\begin{array}{c} A:1,0,0,0;\;\;C:0,1,0,0;\;\;G:0,0,1,0;\\ T:0,0,0,1;\;\;N:0,0,0,0 \end{array}$$

**Figure 4.**
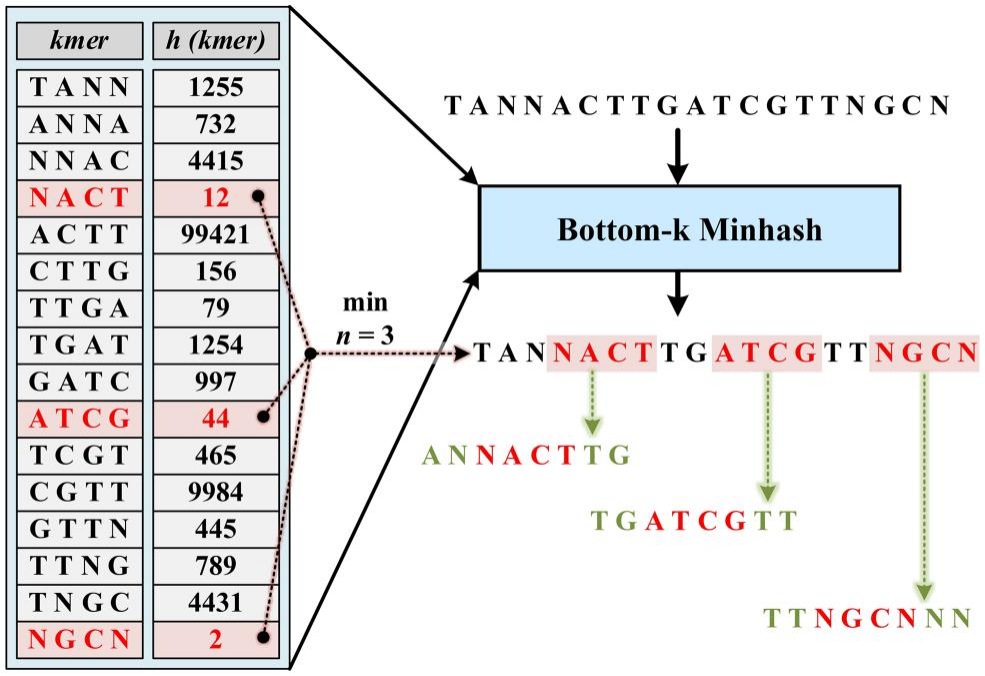
An example of the pre-processing phase, generating three representative fragments. Three minimum 4-mers are selected using Bottom-k MinHash (*h* function). These 4-mers are then extended to form fragments of length 8. One-hot coding is not shown for simplicity.

A pictorial representation detailing this pre-processing workflow is shown in Fig. 4.

**Figure 5.**
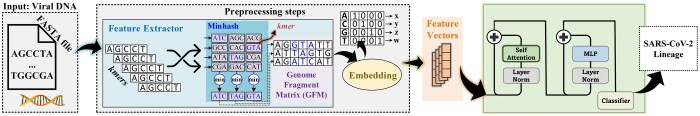
ViTAL algorithm: The input is a sparse (i.e. partially covered) genome in FASTA format. Pre-processing performs feature extraction, composed of MinHash, extension and embedding. Vision Transformer (ViT) receives the feature vectors and outputs the most likely lineage assignments.

2. **Embedding Phase:** Once the one-hot encoding step is completed, each base within the fragments is passed through a base-wise linear layer. This layer computes a representative number for each base, essentially converting the fragments into embeddings. Consequently, *n* numerical vectors of size *f* are generated, representing the embedded fragments.

### 2.4 Data collection

**Figure 6.**
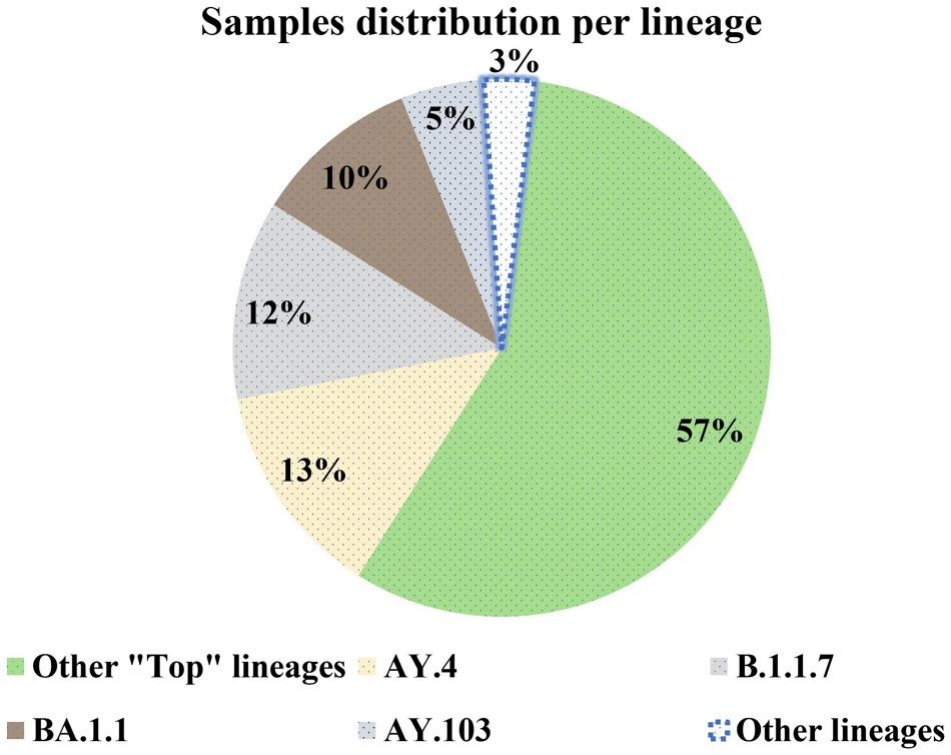
Lineage distribution in the COVID-19 Data Portal; 167 lineages selected for ViTAL training account for 97% of all sequenced genomes in the portal.

A comprehensive dataset used in this study was collected from the COVID-19 Data Portal (https://www.covid19dataportal.org/search/sequences). We selected 167 ‘top’ lineages, which are the most prevalent in the portal’s database. These lineages collectively account for 97% of the sequenced genomes in the portal (Fig. 6), which presumably indicates their global ubiquity.

For each of these 167 lineages, we selected 1024 complete genomes which represent SARS-CoV-2’s genetic diversity. We further partitioned the genome data to facilitate the model training, validation, and testing. The dataset was split into an 80% training set, used primarily for training the neural network. The remaining 20% was equally divided between a validation set and a test set. The validation set assists in tuning and refining the model during the training phase, while the test set is reserved for the final evaluation, ensuring an unbiased assessment of the model’s performance on unseen data. The division of genome data into datasets is sequence release time driven. Specifically, the oldest 80% of the dataset is used for training, 10% next are used for validation, while the newest 10% comprise the test set.

### 2.5 Training

#### 2.5.1 Model architecture and hyperparameters

The primary aim of our training process was to train the neural network to accurately assign lineages, using pre-processed complete genomes as an input. We built several models, each with a different number of transformer encoder layers and different parameters.

To account for the sequencer-dependant coverage bias, DNA reads were extracted from selected positions within each genome in the training set. The read position selection was guided by probabilities derived from Dirichlet distribution (Bishop and Nasrabadi 2006). The Dirichlet distribution is commonly used to model multivariate categorical data and is frequently adopted as a prior distribution in Bayesian inference. Specifically, the symmetric Dirichlet distribution, where all parameters are equal, reflects situations where no prior information is available to favor one component over another. Using the Dirichlet distribution allowed us to account for potential coverage biases introduced by various sequencer platforms. Consequently, the number of DNA reads selected for training was consistent with a predetermined coverage level. Any bases not covered by a read were replaced with the base N, representing ambiguity.

These sparse (i.e. partially covered) complete genomes were then used as input to ViTAL, as described in Section 2. Each processed fragment is 256 bases long, extended from a 16-mer.

We set the value and key dimensions (which determine the size of the query and key matrices) to 256. All models are equipped with eight attention heads. The number of parameters in each model was determined by its encoder depth. The nonlinear activation function is implemented by ReLU (Li and Yuan 2017). The model weights were initialized using the Glorot normal initializer (Glorot and Bengio 2010). We used categorical cross-entropy loss function (Gneiting and Raftery 2007), especially efficient in classification tasks. For optimization, we employed the Adam optimizer with weight decay (Loshchilov and Hutter 2018), owing to its established track record with transformer-based algorithms. The learning process initiated with a rate of 0.01; however, if no improvement in the validation loss was observed over 10 consecutive epochs, the learning rate was halved. To ensure model robustness and guard against overfitting, we employed a dropout rate of 0.2 (Srivastava *et al.* 2014) and complemented it with L2 regularization (λ=0.0001) (Loshchilov and Hutter 2017).

We adopted a methodical approach to choosing the hyperparameters for our models, deploying a grid search to systematically explore the parameter space. This allowed us to identify the optimal set of hyperparameters that yielded the best performance in the lineage assignment task.

#### 2.5.2 Computational infrastructure

For the network training we used Intel Core i7-12700 computer with 128 GB DRAM, and NVIDIA RTX A5000 GPU with 24 GB GDDR.

## 3 Evaluation

### 3.1 Evaluation setup

To simulate the low-coverage sequencing and to account for the sequencer-induced coverage bias, we used sequencing simulators. In particular, we used the ART simulator (Huang *et al.* 2012) to simulate Illumina and 454 Pyrosequencing single-end reads. We used pbsim2 (Ono *et al.* 2021) to simulate PacBio reads. Lastly, we used NanoSim (Yang *et al.* 2017) to simulate Oxford Nanopore Technologies (ONT) reads. The coverage level is a parameter that can be set when running those sequencer simulators.

We aligned the simulated reads to the SARS-CoV-2 Wuhan reference genome using BWA-MEM (Li 2013), following which we applied a neural network based variant calling tool DeepVariant (Poplin *et al.* 2018) to generate VCF files. We then produced a complete sparse genome by replacing variants in the reference and substituting uncovered bases with N’s.

We compare ViTAL against two state of the art lineage assignment and classification tools, UShER (Turakhia *et al.* 2021) and Kraken2 (Wood *et al.* 2019).

We use the *top-n accuracy* as ViTAL assignment accuracy criterion. Top-*n* accuracy quantifies the likelihood of the *correct* assignment [i.e. the lineage to which the query genome truly belongs to (Since the COVID-19 Data Portal assigns all sequenced genomes in our dataset to their respective lineages, the correct assignments are known)] appearing among the *n* most probable results (i.e. *n* lineages output by the network at the top of the descending-order probability list). Specifically, we calculate and present ViTAL’s top-1 accuracy when comparing ViTAL accuracy against state of the art. We use top-5 accuracy when analyzing ViTAL’s ability to identify novel lineages and associate them with their closest relatives.

### 3.2 Accuracy as a function of coverage

In this experiment, we evaluate ViTAL accuracy in assigning sequenced genomes to known lineages (i.e. the lineages ViTAL was trained on) and compare it with that of UShER and Kraken2. Accuracy is evaluated as a function of coverage.

COVID-19 Data Portal assigns all sequenced genomes in our dataset to their respective lineages. Hence the correct assignments are known and can be used to calculate the placement accuracy of all three evaluated solutions.

**Figure 7.**
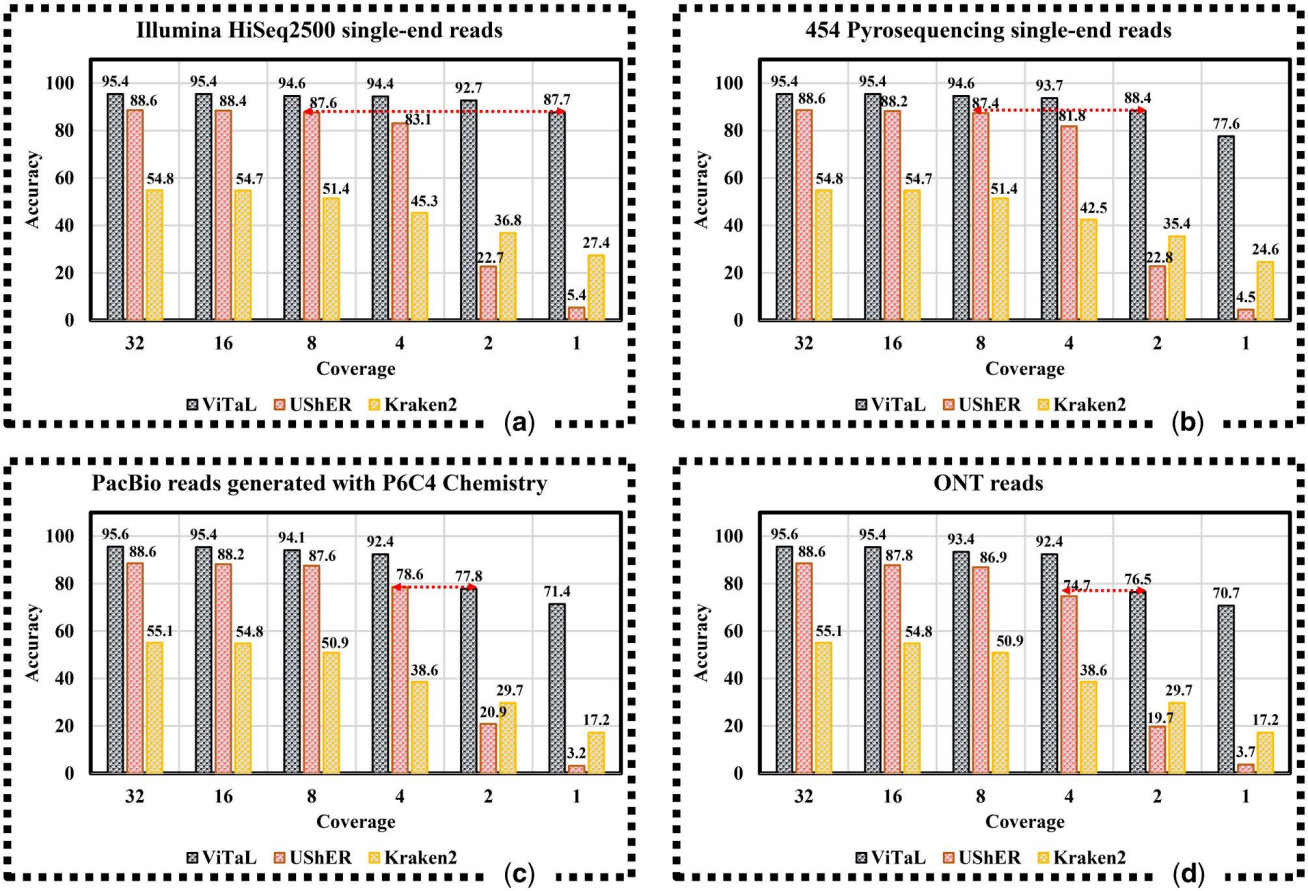
Accuracy of ViTAL, UShER, and Kraken2 as function of sequencing coverage. (a) HiSeq 2500 single-end reads generated by ART (Huang *et al.* 2012), (b) 454 Pyrosequencing single-end reads generated by ART (Huang *et al.* 2012), (c) PacBio reads generated by pbsim2 (Ono *et al.* 2021) with P6C4 chemistry, (d) ONT reads simulated by NanoSim (Yang *et al.* 2017). Horizontal dash arrows show the coverage levels at which ViTAL accuracy is on par with UShER.

Figure 7 shows the lineage assignment accuracy of ViTAL, UShER and Kraken2 as a function of coverage, for SARS-CoV-2 genomes assembled from Illumina HiSeq 2500 single-end reads, 454 Pyrosequencing single-end reads, PacBio reads produced with P6C4 chemistry, and ONT reads respectively.

ViTAL consistently maintains higher level of accuracy across all sequencing platforms and coverage levels. Second to ViTAL is UShER for coverage levels of 4× and higher. For lower coverage levels, Kraken2 outperforms UShER. When applied to genomes assembled using Illumina HiSeq 2500 single end reads, ViTAL achieves at 1× coverage the accuracy level (87.7%) comparable to UShER accuracy at 8× coverage (87.6%) (Fig. 7a). Similarly, for 454 Pyrosequencing single-end reads, ViTAL achieves at 2× coverage the accuracy level (88.4%) comparable to UShER accuracy at 8× coverage (87.4%) (Fig. 7b). Notably, for ONT reads (featuring higher indel rates), ViTAL achieves at 2× coverage the accuracy level (76.5%) slightly higher than UShER accuracy at 4× (74.7%) (Fig. 7d).

### 3.3 Assignment of new mutations to the closest lineages

The last section showed that ViTAL can accurately assign SARS-CoV-2 genomes belonging to lineages ViTAL was trained on, even if the coverage is very low. However, a question arises: can ViTAL confidently assign newly sequenced genomes which come from novel lineages, i.e. lineages not discovered before and hence not involved in ViTAL training?

To evaluate ViTAL’s ability to correctly assign newly sequenced genomes to their closest ancestors, we designed the following experiment. The scenario of novel lineage emergence is simulated by selecting a past date and treating all SARS-CoV-2 lineages discovered until that date as known, and those discovered past that date as novel (not yet discovered). We selected 1 October 2021, as such a date.

We selected from the COVID-19 Data Portal the lineages with at least 512 complete genome samples. There are 77 such lineages predating 1 October 2021. We consequently applied the genomes of these 77 known lineages to train ViTAL.

COVID-19 Data Portal also contains 110 such lineages discovered after 1 October 2021. This lineage set emulates the novel lineages in our experiment.

Since this experiment targets placing new mutations, we assume that there are no ‘right’ answers, i.e. the correct assignments are unknown. Therefore, we applied UShER (Turakhia *et al.* 2021) to place the genomes of the novel lineage set on SARS-CoV-2 phylogenetic tree (https://hgdownload.soe.ucsc.edu/goldenPath/wuhCor1/UShER_SARS-CoV-2/) and used these results as a ‘golden’ reference, which means that ViTAL top-*n* results are considered correct if they contain the UShER result.

**Figure 8.**
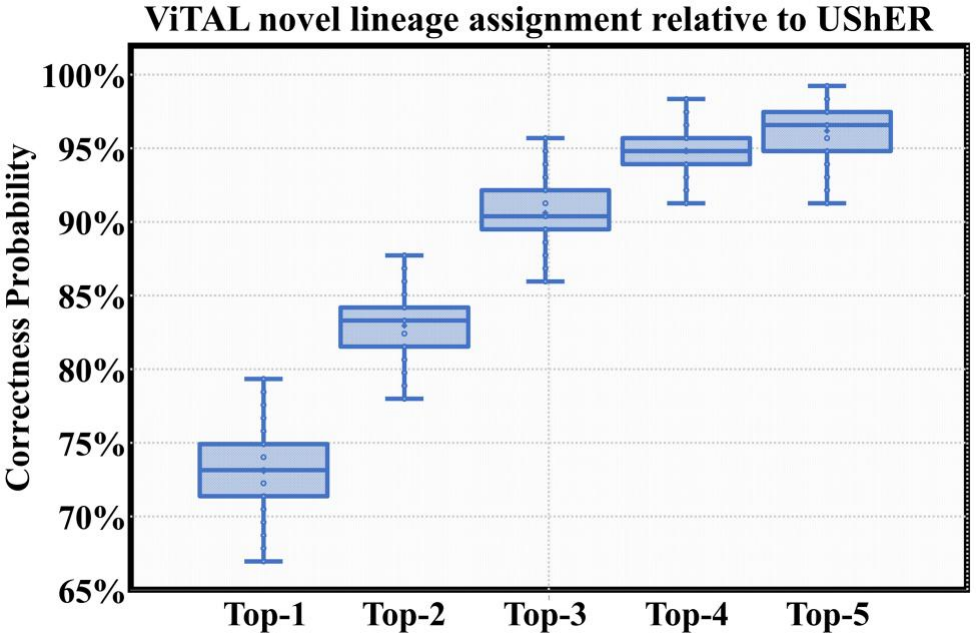
ViTAL accuracy in newly released (as of 10/1/2021, 110 new lineages) genomes assigning; 100% means ViTAL assignment is identical to that of UShER.

Figure 8 presents the distributions of ViTAL results correctness probability for several values of n (top-1 through top-5). The probability of top-1 ViTAL results being correct (i.e. match exactly UShER’s results) across all novel lineages is 73.1%. This figure grows to 96.3% if we consider top-5 results.

These results show that ViTAL’s performance in placing newly sequenced genomes which come from the novel lineages not used in training closely follows UShER’s performance.

### 3.4 Identification of new mutations using ViTAL

In the last section, we show that ViTAL’s performance in placing newly sequenced genomes which come from the novel lineages not used in training, closely follows that of UShER. A follow-up question is: is there a way to ascertain that newly assigned genomes indeed belong to novel (previously undiscovered) lineages?

To answer this question, we further explore the results of the experiment presented in the last section. Specifically, we analyze the top-1 probability values obtained in the experiment. To emphasize, the focus of this new analysis is not on tracing the probability of the top-*n* ViTAL results to match the ancestor node suggested by UShER, but on the absolute value of such top probability.

**Figure 9.**
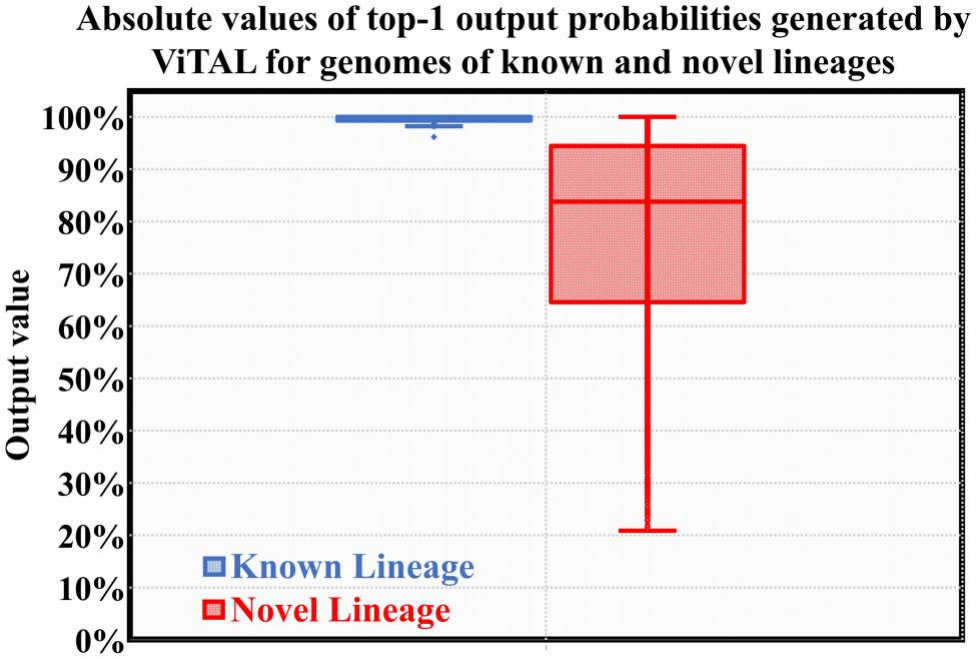
Absolute values of top-1 probabilities for (left box plot) genomes of known lineages ViTAL trained on, and (right box plot) genomes belonging to the novel (as of 10/1/2021) lineages.

The results are presented in Fig. 9. The absolute values of top-1 probabilities of known genomes (i.e. genomes belonging to the 77-lineage set ViTAL was trained on) are shown in blue on the left. The probability levels of the top-1 results of the novel genomes (belonging to lineages discovered after 1 October 2021) are shown in red on the right. While the top-1 results of the known genomes are very close to 100% (reflecting ViTAL confidence in its inference results), the top-1 probabilities of the novel genomes tend to be significantly lower. These two groups can therefore be differentiated by setting a top-1 probability threshold and considering the top-1 probability of each inference result against such threshold.

As an example, defining the threshold at 90% in our settings allows differentiating the novel genomes from the known ones with the success rate of 85.7%. This result suggests that ViTAL cannot only confidently assign a newly sequenced genome of a novel lineage, but also identify a new (previously undiscovered) mutation with a high level of confidence.

### 3.5 Model parameters exploration

**The impact of ViT model depth**. The model depth, i.e. the number of encoder layers, is one of the main parameters of ViT, which strongly affects the computational complexity, the memory requirements and the latency. Therefore, we investigate the impact of the number of encoders in ViTAL ViT model on its assignment accuracy. We vary the number of encoder layers during the training phase, continuing the process until the validation loss reached a stable point.

**Figure 10.**
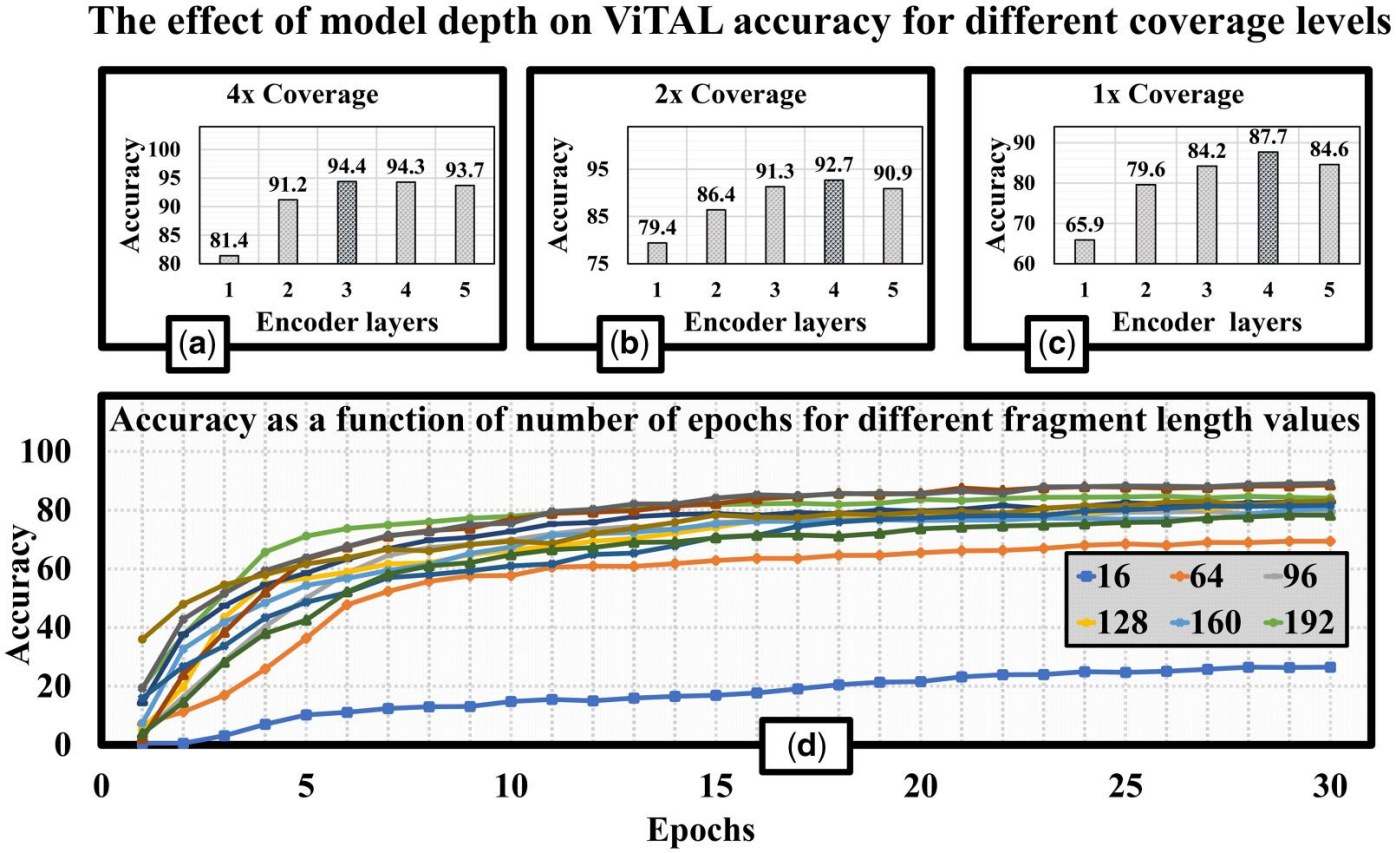
(a)–(c) Accuracy as a function of the number of encoder layers for different coverage levels (4×, 2×, and 1×) for Illumina HiSeq2500 single-end reads; (d) ViTAL accuracy as a function of the fragment length.

The results are presented in Fig. 10a–c, which depicts accuracy as function of the model depth (in encoder layers) for different coverage levels (4×, 2×, and 1×) for Illumina HiSeq2500 single-end reads. The results suggest that a deeper, more intricate model structure is beneficial for enhancing performance, particularly at lower coverage rates. However, 3–4 encoder layers suffice to reach the optimal accuracy. The insight therefore is that ViTAL does not require overly deep and expensive (in terms of compute and memory resources) model to achieve the best accuracy results.

**Fragment length**. The purpose of this study is evaluating the impact of the fragment length on ViTAL accuracy. We trained a single encoder network for 30 epochs with varying fragment length, beginning with a fragment of the length of a single kmer and increasing it to the point where accuracy improvement saturates.

The results are shown in Fig. 10d. Without extending the kmer, the accuracy is very limited (does not exceed 30%). The accuracy improvement beyond the fragment of 256 bases is negligible: increasing the fragment length from 256 to 448 contributes only 2% while the training and inference time grow by 1.7× and 1.4× respectively.

**MinHash Impact**. The role of MinHash in ViTAL is selecting the genome fragments that optimally represent the input sample. To quantitatively evaluate the importance of this mechanism, we tried replacing MinHash fragment selection by a random one. The latter approach led to a significant decrease in model accuracy, which did not surpass 10% regardless of the coverage levels, confirming the critical importance of MinHash in our approach.

## 4 Discussion and conclusions

Several state-of-the-art classifiers (such as Kraken and Clark) use kmer matching for classification. However, these tools face limitations, including the following. First, the exact kmer matching tools may fail to classify erroneous DNA reads. When an edit (replacement or indel) occurs in a kmer, such kmer will not match in the database, which may cause the DNA read to be unclassified and discarded. An example of such classification is presented in (Jahshan *et al.* 2023), where Kraken2 is shown to achieve 64% sensitivity when classifying kmers sourced from PacBio DNA reads with 10% error rate. Second, kmer matching tools are not very efficient in SARS-CoV-2 lineage placement, where the differences between genomes of separate lineages are quite limited. Kraken2 is shown to achieve 55.1% accuracy for 32× coverage, dropping to 17.2% accuracy for 1× coverage for PacBio DNA reads.

LC-WGS offers a time and cost-efficient approach to sequencing large numbers of samples. Arguably the main challenge of this method is the potential reduction in genomic detail, which adversely affects the accuracy of the genome analysis. ViTAL successfully resolves this issue by enabling highly accurate lineage assignment even at coverage rates well below the consensus. This not only streamlines the lineage assignment of quickly mutating viral genomes such as SARS-CoV-2 by reducing dependency on deep sequencing, but also opens doors for broader genomic evaluations. By reducing the need for high-coverage sequencing, ViTAL helps to democratize the evolutionary analysis and genome surveillance of quickly mutating viral pathogens in low-cost and low-quality settings.

Our analysis shows that high accuracy assignment achieved by ViTAL can be attributed to the ability of deep neural networks to learn from broad genomic patterns rather than specific sites within the genome. Thus, even at low coverage, where some important information might be missing, ViTAL can effectively predict the lineage with a high degree of accuracy.

The relationship between model complexity and performance was explored. One of our key findings is that as sequence coverage decreased, the network depth needed to increase to achieve optimal performance. However, excessively increasing the model depth across all coverage rates led to suboptimal performance. This performance drop is due to the model overfitting, which reduces its generalization capability required to accurately assign the unseen test data.

Importantly, we show that ViTAL can be efficiently applied to preliminary assignment of new variants. ViTAL was able to accurately predict the most relevant (closest family members) lineages when classifying genomes of novel lineages (unknown at the time of ViTAL training). Moreover, ViTAL provides the ability to quantitatively differentiate novel lineages by analyzing the levels of the top-1 probability generated by the network.

To summarize, ViTAL enables application of LC-WGS to accurate SARS-CoV-2 lineage assignment and possibly detection of new mutations and novel lineages. ViTAL’s low coverage-tolerant, low cost, rapid and accurate operation potentially makes it a useful tool in our society’s pandemic preparedness.

## Author Biographies

**Zuher Jahshan** is a PhD candidate at the Faculty of Engineering, Bar Ilan University, Israel. He received his MSc and BSc degrees in the Faculty of Electrical and Computer Engineering from Technion, Israel. His research focuses on improving the efficiency of bioinformatic applications. This is achieved primarily through the development of innovative algorithms and the exploration of new computer architectures that can facilitate faster data processing and analysis.

**Leonid Yavits** is with the Faculty of Engineering, Bar-Ilan University, Israel. He received his MSc and PhD degrees in electrical engineering from Technion, Israel. Leonid is a serial entrepreneur who was involved in co-founding and successful management (from a concept to M&A) of several start-ups in the field of ASICs. His research interests include bioinformatics, domain specific accelerators, and processing in memory.
